# Housekeepers, Cooks, and the Death-Rate

**Published:** 1918-04-27

**Authors:** 


### HOUSEKEEPERS, COOKS, AND THE DEATH-RATE.

The Food Controller is well advised to do all he can to make the rationing of food supplies not only as convenient but as popular as possible. It is urged that the majority of people, for instance, are agreeably surprised by the effect of rationing on their own health. It has even been claimed that it has improved the figure and the personal appearance of a majority of His Majesty's lieges. If so, it points a moral which may have a material effect for good on the life and health after the war of many who from want of occupation, the possession of an abundance of this world's goods, or inclination, kill time, as they call it, by abstaining from work, idling to their heart's content, and indulging in the greed for rich foods.

However true or false these effects of rationing ultimately may prove to be, there are other factors which are gravely affecting the comfort and health of no inconsiderable number of people. Rationing has involved a material alteration in the dieting, and in the kinds and quality of foodstuffs available. These changes have told adversely in the case of our elders, and of old people especially, who are suffering from serious discomfort in consequence, and maybe worse ills. We believe that much of the trouble, and all the danger, would be obviated, if British people had the good fortune to be able to hand over the cooking of their food to an artist in its preparation for the table, such as the French cook usually is. The French housewife is proverbially of the best. She is thorough, practical, knowledgeable, and economical. Nothing the world has ever experienced, probably, has made these qualities of higher value than the rationing of food in this country to-day.

The bedrock fact of some serious evils threatened by rationing in the present conditions is, as members of the medical profession know, that the number of workers who suffer from being improperly fed is increasing. The effect on these workers, on whom in many cases the maintenance and upkeep of the home depend, may be disablement for work, and an increasing mortality. These results can be directly brought about by bad dieting and worse cooking, due to a want of cooks of character and enough brains to study the new conditions of our food supplies, their quality, constituents, and preparation for the table. The old gibe that God sends the food and the devil sends the cooks may have a tragic bearing in the present world's war. If we are to judge the Germans and many of their allies by their actions and conduct we must, in justice to their handiwork, regard them as emissaries of the Prince of Darkness. And we make bold to say, from cases which have come to our knowledge, and the consequences to the health of their masters especially, due to the default of careless and obstinately ignorant cooks, that the cook who fails to adapt herself by the acquirement of the necessary knowledge, the following of precise instructions and the shedding of her old extravagant and impracticable notions, is a German in action. For every cook who fails to adapt her methods to the needs of the present system of rationing is not only not doing her bit in this war-time, but she is in fact working for his Satanic majesty by diminishing the vital force of English men and women in their struggle for victory and the triumph of good over evil.

The moral of the present experience and teaching in food matters is that mistresses and their cooks, if they are to do their duty during the war, must have, as never before in their lives, the highest sense of their individual responsibility and duty towards the households of which they are members, for whose health and output in work and energy they may be in fact largely responsible. What a terrible fate to suffer oneself to become a material cause in losing the war, by diminishing the power or causing the permanent enfeeblement or death of those whose health it is our duty and charge to safeguard and maintain! Such, however, may be the fate of heads of households and cooks who by their default cause the breadwinner to fail in his maximum output of energy and work each day. Is there any conduct on their part better calculated to hamper the triumph of their nation and its Allies in this terrible war?

